# Supplementary material for: Do Elderly Patients with Atrial Fibrillation Have Comparable Ablation Outcomes Compared to Younger Ones? Evidence from Pooled Clinical Studies
Source: J Clin Med. 2022 Jul 31;11(15):4468. doi: 10.3390/jcm11154468 (PMC9369410; doi:10.3390/jcm11154468)
Supplement: Supplementary file 1 [file jcm-11-04468-s001.zip › jcm-1790234-Supplementary.pdf]

Journal of
Clinical Medicine

MDPI

Supplementary Table S1. Quality assessment of eligible studies according to the Newcastle-Ottawa Quality Assessment Scale.

		Selection				Comparability	Outcome			Total Stars
First Author	Year	Representativeness of the Exposed Cohort	Selection of the Nonexposed Cohort	Ascertainment of Exposure	Demonstration that Outcome of Interest Was Not Present at Start of Study	Comparability of Cohorts on the Basis of the Design or Analysis	Assessment of Outcome	Was Follow-Up Long Enough for Outcomes to Occur	Adequacy of Follow-Up of Cohorts	
Natale [10]	2021	☆	☆	☆	☆	☆☆	☆	☆	☆	9
Vermeersch [6]	2021	☆	☆	☆	☆	☆☆	☆	☆	☆	9
Sciarra [11]	2021	☆	☆	☆	☆	☆☆	☆	☆	☆	9
Hartl [12]	2021	☆	☆	☆	☆	☆☆	☆	☆		8
Zhou [13]	2020	☆	☆	☆	☆	☆☆	☆	☆		8
Kanda [14]	2019	☆	☆	☆	☆	☆☆	☆	☆	☆	9
Fink [15]	2019	☆	☆	☆	☆	☆☆	☆	☆	☆	9
Zhang-1[16]	2019	☆	☆	☆	☆	☆☆	☆		☆	8
Heeger [17]	2019	☆	☆	☆	☆	☆☆	☆	☆		8
Romero [18]	2019	☆	☆	☆	☆	☆☆	☆	☆		8
Abdin [19]	2019	☆	☆	☆	☆	☆☆	☆	☆	☆	9
Zhang-2 [20]	2019	☆	☆	☆	☆	☆☆	☆	☆		8
Tscholl [21]	2018	☆	☆	☆	☆	☆☆	☆	☆		8
Moser [22]	2017	☆	☆	☆	☆	☆☆	☆	☆	☆	9
Abugattas [23]	2017	☆	☆	☆	☆	☆☆	☆	☆	☆	9
Kautzner [24]	2017	☆	☆	☆	☆	☆☆	☆	☆	☆	9
Bunch-2 [25]	2016	☆	☆	☆	☆	☆☆	☆	☆	☆	9
Lioni [26]	2014	☆	☆	☆	☆	☆☆	☆	☆	☆	9
Santangeli [27]	2012	☆	☆	☆	☆	☆☆	☆	☆	☆	9
Hao [28]	2012	☆	☆	☆	☆	☆☆	☆		☆	8
Bunch-1 [29]	2010	☆	☆	☆	☆	☆☆	☆	☆	☆	9
Kusumoto [30]	2009	☆	☆	☆	☆	☆☆	☆	☆	☆	9
Bhargava [31]	2004	☆	☆	☆	☆	☆☆	☆	☆	☆	9

Note: ☆ represents a star, and ☆☆ represents two stars

Supplementary Table S2. Quality assessment of the single-arm studies according to the Institute of Health Economics checklist.

First author	Year	Study Objective	Study Design	Study Population	Intervention and Cointervention	Outcome Measures	Statistical Analysis	Results and Conclusions	Quality Score
Liu [32]	2022	1	3	3	2	3	1	5	18
Akhtar [33]	2020	1	1	3	2	3	1	5	16
Metzner [34]	2016	1	2	3	2	3	1	4	16
Corrado [35]	2008	1	2	3	2	3	1	4	16

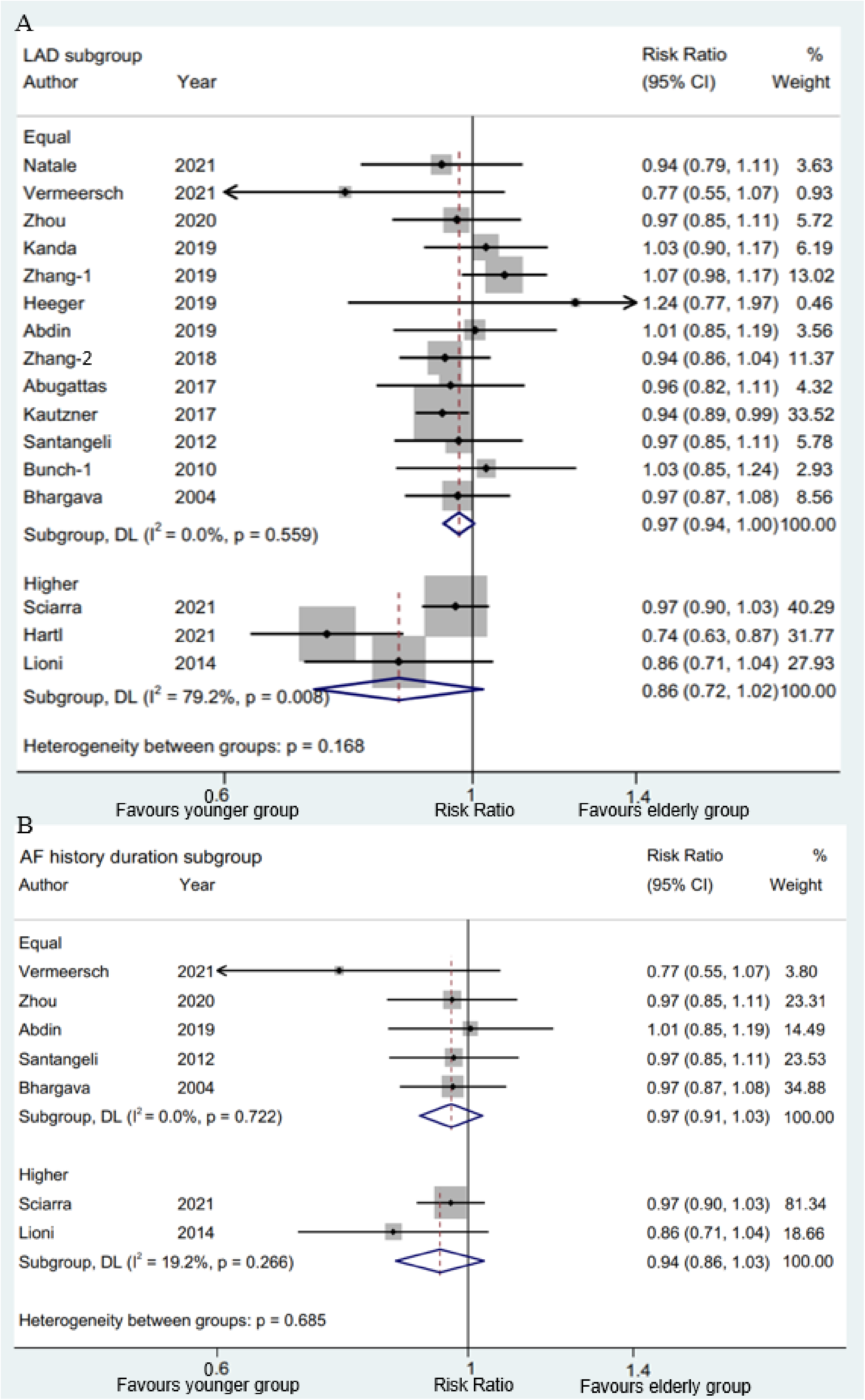
A
LAD subgroup
Author Year
Risk Ratio (95% CI)
% Weight
Equal
Natale 2021 0.94 (0.79, 1.11) 3.63
Vermeersch 2021 0.77 (0.55, 1.07) 0.93
Zhou 2020 0.97 (0.85, 1.11) 5.72
Kanda 2019 1.03 (0.90, 1.17) 6.19
Zhang-1 2019 1.07 (0.98, 1.17) 13.02
Heeger 2019 1.24 (0.77, 1.97) 0.46
Abdin 2019 1.01 (0.85, 1.19) 3.56
Zhang-2 2018 0.94 (0.86, 1.04) 11.37
Abugattas 2017 0.96 (0.82, 1.11) 4.32
Kautzner 2017 0.94 (0.89, 0.99) 33.52
Santangeli 2012 0.97 (0.85, 1.11) 5.78
Bunch-1 2010 1.03 (0.85, 1.24) 2.93
Bhargava 2004 0.97 (0.87, 1.08) 8.56
Subgroup, DL (I² = 0.0%, p = 0.559) 0.97 (0.94, 1.00) 100.00
Higher
Sciarra 2021 0.97 (0.90, 1.03) 40.29
Hartl 2021 0.74 (0.63, 0.87) 31.77
Lioni 2014 0.86 (0.71, 1.04) 27.93
Subgroup, DL (I² = 79.2%, p = 0.008) 0.86 (0.72, 1.02) 100.00
Heterogeneity between groups: p = 0.168
0.6
1
1.4
Favours younger group
Risk Ratio
Favours elderly group
B
AF history duration subgroup
Author Year
Risk Ratio (95% CI)
% Weight
Equal
Vermeersch 2021 0.77 (0.55, 1.07) 3.80
Zhou 2020 0.97 (0.85, 1.11) 23.31
Abdin 2019 1.01 (0.85, 1.19) 14.49
Santangeli 2012 0.97 (0.85, 1.11) 23.53
Bhargava 2004 0.97 (0.87, 1.08) 34.88
Subgroup, DL (I² = 0.0%, p = 0.722) 0.97 (0.91, 1.03) 100.00
Higher
Sciarra 2021 0.97 (0.90, 1.03) 81.34
Lioni 2014 0.86 (0.71, 1.04) 18.66
Subgroup, DL (I² = 19.2%, p = 0.266) 0.94 (0.86, 1.03) 100.00
Heterogeneity between groups: p = 0.685
0.6
1
1.4
Favours younger group
Risk Ratio
Favours elderly group

Supplementary Figure S1. Forest plot of subgroup analysis of the rates of freedom from AF between elderly and younger groups in terms of LAD and AF history duration. (**A**) Subgroup analysis of the rates of freedom from AF between elderly and younger groups in terms of LAD showed no significant difference between the equal and higher subgroups (p = 0.168) [6,10-14,16,17,19,20,23,24,26,27,29,31]; (**B**) subgroup analysis of the rates of freedom from AF between elderly and younger groups in terms of AF history duration showed no significant difference between the equal and higher subgroups (p = 0.685) [6,11,13,19,26,27,31]. LAD: left atrial diameter; AF: atrial fibrillation.

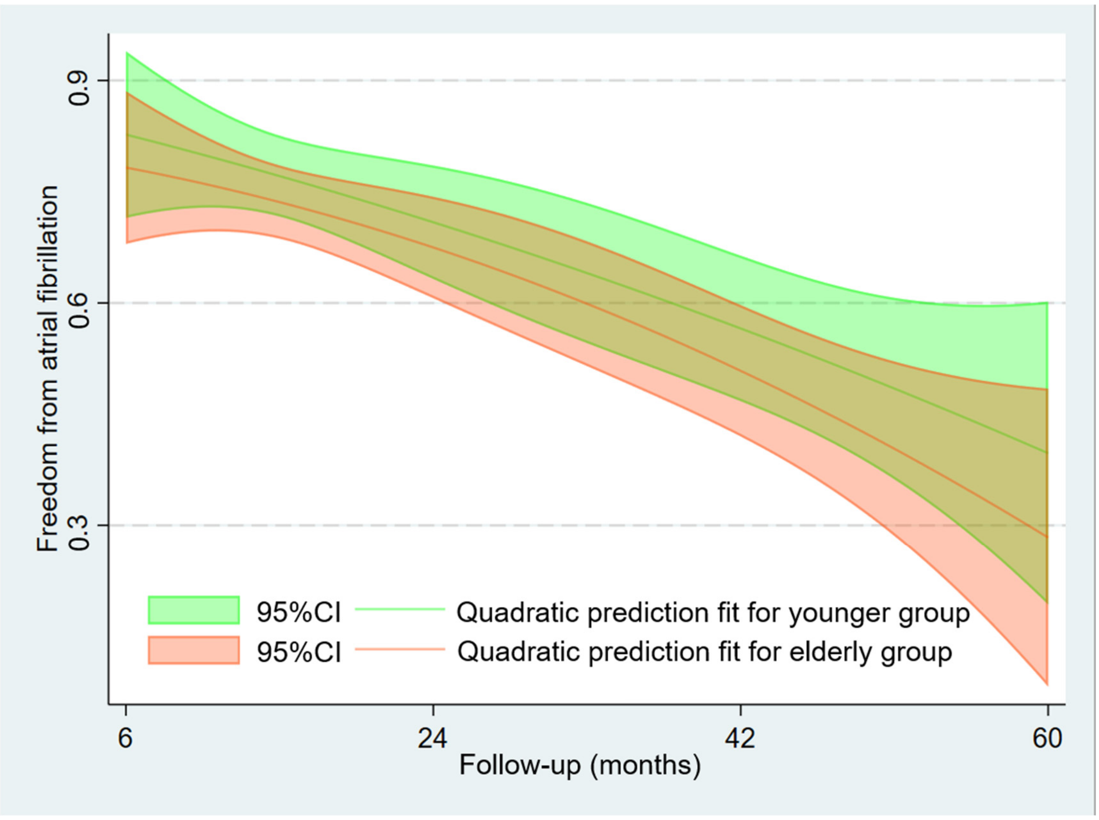

Supplementary Figure S2. Quadratic prediction fit plot with confidence intervals between the follow-up time and the rates of freedom from AF for the elderly group and the younger group. Overlapping the quadratic prediction fit plot with the confidence interval for elderly and younger groups to visualize the difference in rates of freedom from AF between the two groups. AF: atrial fibrillation; CI: confidence interval.

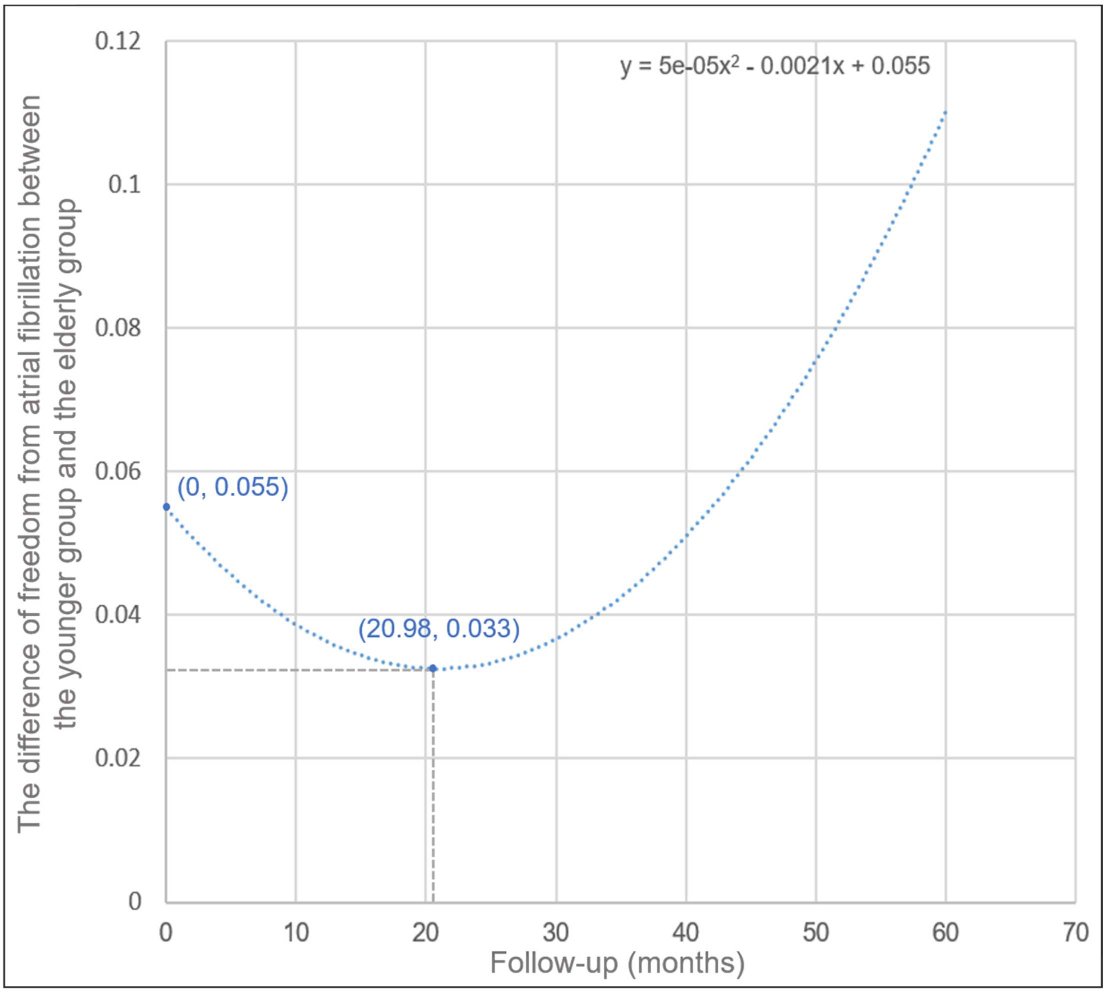

Supplementary Figure S3. The relationship of the follow-up time and the difference of freedom from AF between the younger group and the elderly group. The difference monotonically decreased in the interval of 0 to 20.98 months, while the difference monotonically increased in the interval of ≥ 20.98 months. AF: atrial fibrillation.

Supplementary Table S3. Pooled incidence of safety outcomes in the elderly group.

Safety Outcomes	Numbers of Study	Pooled Incidence	95% CI	I^2 (%)	*p* Value
Cerebrovascular events	24	0.00	0.00–0.01	30.40	0.000
Serious hemorrhage complications	22	0.00	0.00–0.01	15.98	0.000
Phrenic nerve injury	14	0.01	0.00–0.02	77.25	0.011
All-cause death	16	0.01	0.00–0.02	83.82	0.050

Notes: Cerebrovascular events include stoke or transient ischemic attack (TIA); serious hemorrhage complications include hemothorax, perforation, tamponade, or major bleeding.
